# Notices

**Published:** 1873-04

**Authors:** 


### NOTICES.

### *American Medical Association.*

OFFICE OF PERMANENT SECRETARY,
1400 Pine Street, S. W. cor. Broad, Philadelphia.

The Twenty-Fourth Annual Session will be held in St. Louis, Mo., May 6, 1873, at 11 A. M.

The following committees are expected to report:

On Cultivation of the Cinchona Tree. Dr. Lemuel J. Deal, Philadelphia, Pa., Chairman.

On Measures to Prevent the Extension of Diseases of Inferior Animals to Man, and the Sanitary Measures to Arrest the Progress of such Diseases in Animals. Dr. A. W. Stein, New York, N. Y., Chairman.

On the Treatment of Fractures. Dr. Lewis A. Sayre, New York, N. Y., Chairman.

On Gunguillia as a Substitute for Quinia. Dr. Wm. Chew Van Bibber, Baltimore, Md., Chairman.

On Gynæcology. Dr. Montrose A. Pallen, St. Louis, Mo., Chairman.

On the Renewal of Prescriptions without Authority, and on the Relations of Physicians and Druggists. Dr. R. J. O'Sullivan, New York, N. Y., Chairman.

On Vaccination. Dr. T. N. Wise, Covington, Ky., Chairman.

On Skin Transplantation. Dr. J. Ford Thompson, Washington, D. C., Chairman.

On some Diseases peculiar to Colorado. Dr. John Elsner, Denver, Col., Chairman.

On Correspondence with State Medical Societies. Dr. N. S. Davis, Chicago, Ill., Chairman.

On National Health Council. Dr. Thomas M. Logan, Sacramento, Cal., Chairman.

On Nomenclature of Diseases. Dr. Francis Gurney Smith, Philadelphia, Pa., Chairman.

On American Medical Necrology. Dr. J. D. Jackson, Danville, Kentucky, Chairman.

On Suggestions on Medical Education. Dr. A. M. Pollock, Pittsburgh, Pa., Chairman.

On Medical Education. Dr. William Carson, Cincinnati, Ohio, Chairman.

On Medical Literature. Dr. Austin Flint, New York, N. Y., Chairman.

On Prize Essays. Dr. John S. Moore, St. Louis, Mo., Chairman.

On Plan for better Arrangement of Sections, and more Rigid Examination of Papers offered for Publication. Dr. E. L. Howard, Baltimore, Md., Chairman.

On Ethics. Dr. H. F. Askew, Wilmington, Del., Chairman.

On the Climatology and Epidemics of—

Alabama, Dr. T. C. Osborne; Arkansas, Dr. G. W. Lawrence; California, Dr. W. H. Williams; Colorado, Dr. R. G. Buckingham; Connecticut, Dr. J. C. Jackson; Delaware, Dr. L. P. Bush; District of Columbia, Dr. J. W. H. Lovejoy; Georgia, Dr. G. M. McDowell; Illinois, Dr. David Prince; Indiana, Dr. Dugan Clark; Iowa, Dr. G. M. Staples; Kansas, Dr. Tiffin Sinks; Kentucky, Dr. L. P. Yandell, Sr.; Louisiana, Dr. S. M. Bemiss; Maine, Dr. S. H. Tewksbury; Maryland, Dr. C. H. Ohr; Massachusetts, Dr. E. Cutter; Michigan, Dr. S. H. Douglass; Minnesota, Dr. Charles N. Hewitt; Mississippi, Dr. S. V. D. Hill; Missouri, Dr. T. B. Lester; New Hampshire, Dr. G. A. Crosby; New Jersey, Dr. E. M. Hunt; New York, Dr. Gouverneur M. Smith; North Carolina, Dr. E. Burke Haywood; Ohio, Dr. J. A. Murphy; Oregon, Dr. Horace Carpenter; Pennsylvania, Dr. W. L. Wells; Rhode Island, Dr. Edward T. Caswell; South Carolina, Dr. M. Simmons; Tennessee, Dr. W. K. Bowling; Texas, Dr. S. M. Welch; Vermont, Dr. G. B. Bullard; Virginia, Dr. H. A. Claiborne; West Virginia, Dr. H. W. Brock; Wisconsin, Dr. J. K. Bartlett.

Physicians desiring to present papers before the Association should observe the following rule:

"Papers appropriate to the several sections, in order to secure consideration and action, must be sent to the Secretary of the appropriate section at least one month before the meeting which is to act upon them. It shall be the duty of the Secretary to whom such papers are sent, to examine them with care, and, with the advice of the Chairman of his section, to determine the time and order of their presentation, and give due notice of the same."

Officers of Sections.

*Chemistry and Materia Medica.*—Drs. R. E. Rogers, Philadelphia, Pa., Chairman; Ephraim Cutter, Boston, Mass., Sec.

*Practice of Medicine and Obstetrics.*—Drs. D. A. O'Donnell, Baltimore, Md., Chairman; Benjamin F. Dawson, New York, N. Y., Sec.

*Surgery and Anatomy.*—Drs. Edward Warren, Baltimore, Md., Chairman; W. F. Peck, Davenport, Iowa, Sec.

*Meteorology and Epidemics.*—Drs. George Sutton, Aurora, Indiana, Chairman; Elisha Harris, New York, N. Y., Sec.

*Medical Jurisprudence, Hygiene and Physiology.*—Drs. S. C. Busey, Washington, D. C., Chairman; A. B. Arnold, Baltimore, M.D., Sec.

*Psychology.*—Drs. Isaac Ray, Philadelphia, Pa., Chairman; John Curwen, Harrisburg, Pa., Sec.

Amendments to be Acted On.

(to constitution.)

*Resolved,* That the United States Marine Hospital Service be placed in the same relative position in the American Medical Association as the Medical Department of the United States Army and Navy.

And that in paragraph 2, of the 2nd section, after the words "army and navy," the words "and the United States Marine Hospital Service" be inserted.

(to by-laws.)

Sec. III.—*Standing Committees.*

That, instead of a report on Medical Education, Medical Literature, and Climatology and Epidemic Diseases, there shall be annually delivered before the Association, at its general meetings, an address in medicine, an address in surgery, and an address in midwifery, or the diseases of children, the lecturers to be appointed this year by the President; afterwards by the Committee on Nominations.

Also, in section 6, after the words, "the chiefs of the bureaus of the army and navy," be inserted "and the supervising surgeon of the United States Marine Hospital Service."

Secretaries of all medical organizations are requested to forward lists of their delegates, as soon as elected, to the Permanent Secretary.

Wm. B. Atkinson, M.D., Permanent Secretary.

No. 1400 Pine Street, Philadelphia.

We cheerfully call attention to the following circular:

**Medical Register and Directory of the United States.**

Work on this important publication, which was delayed by illness, is now resumed with energy, and it will be issued as soon as the vast amount of material can be collected and arranged. The *Register and Directory* will contain as complete a list of the medical men in the United States, with their professional status, as can be obtained by personal application to each, and from other sources.

Also, a complete medical history of each State, including all its medical institutions, societies, etc., and all medical legislation. Nothing will be omitted that will be of possible benefit to the profession. The co-operation of medical men in every section of the country is earnestly solicited in replying to the circulars sent out, and in *giving brief outlines of the history of medical institutions, hospitals, colleges, etc., and of State, and the more important local medical organizations.*

Circulars will be sent out hereafter *by States*, and announced through the medical journals. They *have been sent* to the following States and Territories, viz.: Alabama, Arizona, Arkansas, California, Colorado, Connecticut, Dakotah, Delaware, District of Columbia, Florida, and Georgia. In *March*, they will be sent to Illinois, Indiana, Iowa, Kansas, Kentucky, and Louisiana, and all the remaining territories.

IMMEDIATE ANSWERS are requested, and prompt notification if circulars are not received.

Physicians are particularly requested to make as full replies as possible to the questions in the circular.

☞ Medical Journals please notice.

S. W. BUTLER, M.D., 115 S. Seventh St., Philadelphia.

**To American Publishers and Authors.**

There are many American Books, Pamphlets, Maps, etc., so interesting, important, special and unique, and therefore so deserving of wide recognition, that there would undoubtedly be a considerable demand for them in foreign countries, if public attention were there properly directed to them.

This I believe I am in position to do, and thus open out, in a measure, the market *outside America* for *Original American Literature* by appending to my CATALOGUE OF AMERICAN PERIODICAL LITERATURE a list of *original* American publications (including translations, but *excluding all reprints*).

I should regard such Appendix simply as an experiment which could not be perfected on the first attempt. However, should the reception of this List abroad warrant it, I would, in a second edition, aim at greater completeness.

The value of such List of Books, etc., will be considerably enhanced by an *Index* in which the *subject-matter* of each will be found registered, so that on looking for any *special* subject one would be referred to the various publications treating of the same.

I desire especially to enumerate *original* American publications in the following departments:

American Antiquities, Bibliography, Biography, Education (exclusive of School Books,) Geography, History, Jurisprudence, Languages, Politics, Statistics, and other important matters specially *American.*

Theology (of all Schools and Sects), Philosophy, Medicine and Surgery, Natural History, Chemistry and Pharmacy, Natural Philosophy, Mathematics and Astronomy, Modern Languages and Philology, (exclusive of School Books), Architecture, Engineering, Manufactures, Mechanics, Military and Naval Science, Commerce and Finance, Rural and Domestic Economy, Fine Arts and Music, Secret Societies, Spiritualism, Atlases, Maps and Charts, Views of American Scenery.

Publishers or authors desirous of having their publications inserted in my Catalogue, and in the Index, will please apply to me for the requisite number of blank forms, which must be carefully filled up, and returned to me without delay. E. STEIGER.

New York, 1873, Feb. 5th.

### *Appeal of the Philadelphia Obstetrical Society for Aid in the Formation of a Museum of Distorted Pelves, Obstetrical and Gynæcological Instruments.*

At a recent meeting of the Philadelphia Obstetrical Society, it was decided to establish a Museum, for the collection of deformed and distorted Pelves, and for the preservation of Obstetrical Instruments possessing historical value, or illustrating new methods of treatment.

The Society was led to take this action for these reasons :

1st. No subject at present possesses more interest to the obstetrician than the study of the various deformities of the Pelvis, their mode of production, their influence on the process of parturition, and the principles which should guide the accoucheur when operative interference is deemed necessary.

The Profession is gradually becoming more and more convinced of the influence of contractions, more or less marked, in causing not only tedious and difficult labors, but also in the production of mal-presentations and of many of the accidents of labor.

At present there does not exist any extensive collection of Female Pelves, by which a comprehensive study of this subject can be successfully undertaken. Feeling, therefore, the importance of such observation, the Society proposes to establish a MUSEUM, having this object in view, and would therefore earnestly solicit such specimens of contracted Pelves as may be in the possession of members of the Medical Profession, who may be willing to yield the pleasure of individual possession, in order to assist in forming a collection which will allow a wider and more comprehensive survey of this subject. If the original specimen cannot be sent, casts or photographs are solicited. In certain cases, possessing unusual interest, the Society is prepared to offer a pecuniary recompense, should this be desired.

2nd. Recognizing the fact, that various Instruments, designed for Obstetric Manipulations, or for the performance of operations in Uterine Surgery, having been superseded by new and improved models, now possess only an historical interest, the Society has determined to collect such instruments and preserve them, as illustrating the progress of this branch of our art in America. We would also warmly urge upon the inventors of new or modified instruments, and upon Surgical Instrument Makers in general, the desirability of presenting to the Society specimens of Instruments, Pessaries, and special mechanical contrivances, which they may be desirous of bringing before the Profession.

All objects, whether embraced in the first or second class, will be conspicuously placed in the Museum of the Society, after having been labeled with an explanatory description, and with the name of the donor.

They will also be carefully preserved, open to the inspection of all interested in the support and advancement of Obstetrics and the kindred branches of Medicine.

All objects for the Museum may be sent to the Secretary of the Society, Dr. J. V. Ingham, No. 1342 Spruce Street, Philadelphia, who will at once acknowledge their receipt, and will gladly furnish such additional information as may be desired.

By order of the Society. Wm. Goodell, M.D., President.

Wm. F. Jenks, M.D.,<br>

J. V. Ingham, M.D.,<br>

Horace Williams, M.D., } Committee.

